# Electrochemical Rapid Detection of Methamphetamine from Confiscated Samples Using a Graphene-Based Printed Platform

**DOI:** 10.3390/s23136193

**Published:** 2023-07-06

**Authors:** Florina Truta, Ana-Maria Drăgan, Mihaela Tertis, Marc Parrilla, Amorn Slosse, Filip Van Durme, Karolien de Wael, Cecilia Cristea

**Affiliations:** 1 Department of Analytical Chemistry, Iuliu Hațieganu University of Medicine and Pharmacy, 8 Victor Babes, 400012 Cluj-Napoca, Romania; florina.truta@umfcluj.ro (F.T.); ana.dragan@umfcluj.ro (A.-M.D.); mihaela.tertis@umfcluj.ro (M.T.); 2 A-Sense Lab, University of Antwerp, Groenenborgerlaan 171, 2010 Antwerp, Belgium; marc.parrillapons@uantwerpen.be (M.P.); karolien.dewael@uantwerpen.be (K.d.W.); 3 NANOlab Center of Excellence, University of Antwerp, Groenenborgerlaan 171, 2010 Antwerp, Belgium; 4 National Institute for Criminalistics and Criminology (NICC), Vilvoordsesteenweg 100, 1120 Brussels, Belgium; amorn.slosse@just.fgov.be (A.S.); filip.vandurme@just.fgov.be (F.V.D.)

**Keywords:** methamphetamine, graphene, electrochemical fingerprinting, street samples, water samples

## Abstract

Methamphetamine (MAP) is a highly addictive and illegal stimulant drug that has a significant impact on the central nervous system. Its detection in biological and street samples is crucial for various organizations involved in forensic medicine, anti-drug efforts, and clinical diagnosis. In recent years, nanotechnology and nanomaterials have played a significant role in the development of analytical sensors for MAP detection. In this study, a fast, simple, and cost-effective electrochemical sensor is presented that is used for the sensitive detection of MAP in confiscated street samples with a complex matrix. The optimized screen-printed sensor based on a carbon working electrode modified with graphene demonstrated an excellent limit of detection, good sensitivity, and a wide dynamic range (1–500 μM) for the target illicit drug both for standard solutions and real samples (seized samples, tap water, and wastewater samples). It can detect MAP at concentrations as low as 300 nM in real samples. This limit of detection is suitable for the rapid preliminary screening of suspicious samples in customs, ports, airports, and on the street. Furthermore, the sensor exhibits a good recovery rate, indicating its reliability and repeatability. This quality is crucial for ensuring consistent and accurate results during screening processes.

## 1. Introduction

Currently, drugs of abuse represent a global concern [1]. Amphetamines are the second-most-used illicit drug, after cannabis. Consequently, methamphetamine (MAP) has received much attention as an amphetamine-like stimulant drug [2,3].

MAP is a powerful central nervous system stimulant that can cause mental alertness and increased energy [1,2]. The most common forms of MAP are powder or crystals, and they are consumed by oral route, injecting, or smoking [4]. A lower oral dose of MAP can improve cognitive function, causing effects like positive mood and euphoria and reducing fatigue, but if the consumption is repeated, tolerance can appear and the consumer rapidly becomes dependent [5].

The most concerning effects that appear in the case of chronic use are cardiovascular anomalies, nutritional deficiencies, sleep deprivation, and decreased cognitive functioning [1,3]. As a result, the determination of MAP has attracted much attention [6].

There is a great interest in the detection of this compound, preferably with a cheap and effective method, from various matrices, ranging from seized samples to biological and environmental samples [2]. The most commonly used methods for the detection of MAP nowadays include gas chromatography coupled with mass spectrometry (GC-MS), high-performance liquid chromatography coupled with mass spectrometry (HPLC-MS), capillary electrophoresis (CE), and spectrometry [1]. These methods present some disadvantages, like time-consuming procedures, expensive apparatus, and tedious and complicated sample pre-treatment [1].

Developing a simple and inexpensive electrochemical method for MAP detection that can provide better sensitivity and reduced analysis times, thus facilitating the development of rapid tests that can be useful in real-world scenarios [2,7,8,9].

Nowadays, the substances that are present in the seized samples besides the illicit drug represent a great concern. These substances can be classified as follows: (i) cutting agents or bulk agents, which are used as fillers, (ii) adulterants, which are used to suppress the adverse effects of the drug, to mimic and enhance the desired ones, or to facilitate the administration, and (iii) other drugs of abuse [10].

Illicit drugs are recognized as a group of emerging environmental pollutants and can be detected in environmental matrices such as tap water, wastewater, and surface water as a criminalistics tool for the evaluation of illicit drug consumption and the localization of clandestine laboratories [11,12], MAP being one of the illicit drugs that have undergone such an analysis [13,14]. Furthermore, illicit drug analysis in environmental waters is employed for the evaluation of the effects that these pollutants may have on the local fauna and microbial communities [15]. The gold-standard method employed in this regard is HPLC-MS/MS, but recently sensors and biosensors have begun to represent a convenient alternative [2,11,16,17].

In this study, an extensive investigation was conducted to assess the direct electrochemical transformation of MAP and the impact of the functionalization of planar screen-printed carbon electrodes with different nanomaterials such as graphene and multiwalled carbon nanotubes on the electrochemical signal obtained. Other graphene-based sensors were developed for electrochemical direct detection of MAP, but the platform used was based on glassy carbon electrodes and was suited for assessing illicit drugs from contaminated surfaces such as glass, stainless steel, plastic, and varnished wood [18,19]. This sensor possessed similar analytical performance to the sensor developed in the present study, but it is not portable and was not tested in the matrices assessed in our study, i.e., seized samples and water samples. By evaluating different platforms and different reaction media, we aimed to identify the most suitable ones for detecting the target illicit drug. Specifically, we focused on the current signal recorded after the electrochemical oxidation of the target analyte, which could be applied for the direct and rapid detection of this illicit drug from standard samples and complex real matrices. The choice of platforms incorporating nanomaterials was motivated by their unique properties, such as high surface area, excellent conductivity, and catalytic activity. These characteristics make them promising candidates for enhancing the sensitivity and selectivity of electrochemical drug detection systems. To assess the influence of electrolytic media, we used two different pH values. The pH of the electrolytic medium can significantly affect the ionization state and stability of illicit drugs and their adulterants, thereby influencing the detection process. By examining multiple pH conditions, we gained valuable insights into the performance of the platforms under different physiological and environmental conditions.

Electrochemical methods such as cyclic voltammetry (CV) and square wave voltammetry (SWV) were applied to test the electrochemical behavior of MAP.

The range of concentrations for which there is a linear variation of the analytical signal with the concentration of MAP was determined, and then the selectivity of the sensor towards the target analyte in multicomponent solutions, where it is in combination with other illicit drugs but also with common adulterants/cutting agents, was tested. The validation of the sensor was carried out by field testing, with the help of a portable potentiostat, for MAP detection in real samples (confiscated street samples and water samples), but also by comparing the results with those obtained with conventional methods currently applied in the analysis of illicit drugs, such as GC-MS, GC-FID, FTIR, and Raman. The optimized method has proven its potential for the fast, decentralized screening of suspicious samples captured from the street and environmental waters.

## 2. Materials and Methods

### 2.1. Materials and Instrumentation

All the chemical solvents and substances used in this work were of analytical grade and were used as received from the manufacturer without any further purification. Methamphetamine was purchased from Cayman Chemicals (Ann Arbor, MI, USA), and acetaminophen, benzocaine, caffeine, lactose, procaine, quinine, starch K_2_HPO_4_, KH_2_PO_4_, KCl, HCl, and NaOH were purchased from Merck (Rahway, NJ, USA).

All experiments were performed using a phosphate buffer saline (PBS) solution of 20 mM with 0.1 M KCl as the supporting electrolyte, which was prepared with K_2_HPO_4_ and KH_2_PO_4_, adjusted to the corresponding pH values (7 or 12) with either NaOH or HCl. All aqueous solutions used were prepared with ultrapure water (18.2 MΩ, Adrona B30, Vilnius, Lithuania). Britton Robinson buffer (BR) consists of a mixture of equal concentrations (40 μM) of boric acid, phosphoric acid, and acetic acid that has been titrated to the desired pH (between 7 and 12) with 0.1 M NaOH.

For all the experiments, the electrodes used were custom screen-printed electrodes based on carbon working electrodes (Dropsens—Metrohm, Oviedo, Spain), which had a silver pseudo-reference and a carbon counter electrode. The surface of the working electrodes was functionalized with graphene (GPH) or with multi-walled carbon nanotubes (MWCNTs) (Ø = 4 mm).

The electrochemical experiments (SWV and CV) were performed using an AUTOLAB PGSTAT 302N potentiostat (EcoChemie, Utrecht, The Netherlands) equipped with the associated NOVA 1.10 software. The analysis of the experimental data and the generation of figures were performed using the Origin 8.5 software (OriginLab, Northampton, MA, USA).

### 2.2. Methamphetamine Characterization via CV

An electrochemical characterization of MAP on three different platforms (graphite, GPH, and MWCNTs) was performed by CV using a 0.5 mM MAP solution in PBS (20 mM) with 0.1 M KCl at two pH values (7 and 12). The CV was performed in the potential window from −0.8 V to 1.6 V with a scan rate of 100 mV/s.

### 2.3. Electrochemical Fingerprinting of Methamphetamine/Adulterants by SWV

All electrochemical fingerprints were obtained using SWV with the following parameters: a potential window of 0 to 1.3 V, a step potential of 5 mV, a scan rate of 100 mV/s, an amplitude of 25 mV, and a frequency of 10 Hz. All the solutions used were prepared in PBS or BR buffer.

Firstly, the electrochemical fingerprint of MAP was performed at two pHs (7 and 12) and on three different platforms (graphite, GPH, and MWCNTs) using a 0.5 mM MAP solution. The optimization of pH was done by using CV, SWV, and 100 μM MAP solutions in BR buffers of different pH. The optimization of the scan rate was done by using CV, SWV, and 100 μM MAP solutions in PBS at pH 12. Afterwards, using the optimal pH, scan rate, and platform, the electrochemical fingerprints of (i) 10 adulterants/cutting agents (acetaminophen, benzocaine, caffeine, lactose, procaine, quinine, and starch) in a 0.5 mM solution and (ii) binary mixtures of MAP with each adulterant/cutting agent in a 1:1 ratio (0.5 mM:0.5 mM) were obtained.

### 2.4. Analytical Performance

The analytical performance of the optimized method was evaluated by several parameters: calibration curve, limit of detection (LOD), and limit of quantification (LOQ). The LOD value was estimated based on the signal-to-noise ratio of 3 (S/*n* = 3), and the LOQ represents the lower limit of the dynamic range of MAP concentration tested for the calibration.

### 2.5. Assessment of Real Samples

Finally, the optimized method was applied to real samples consisting of street seized samples, tap, and wastewater samples. The real samples were processed as follows:

(a) Street seized samples: a solution of about 0.3 mg/mL of the suspicious confiscated powders (12 seized samples) was prepared in PBS pH 12. The mixture was thoroughly mixed for 30 s before being used for testing. Thereafter, a 100 uL drop was deposited on the screen-printed electrodes for the electrochemical interrogation. The street samples prepared as mentioned were tested without any other pre-treatment.

The composition of the seized real samples was assessed in the forensic laboratory at NICC using standard methods. The tests aimed to validate the electrochemical sensor, and both the identification and quantification of the samples were performed with gas chromatography-mass spectrometry (GC-MS) and gas chromatography with flame-ionization detector (GC-FID), respectively, which were used as reference methods. At the NICC drugs laboratory, these methods were accredited by the ISO17025 standard and are continuously evaluated through participation in international quality control programs (United Nations Office on Drugs and Crime—UNODC, and European Network of Forensic Science Institutes—ENFSI). Quality is assured with an in-house quality control (QC) sample and participation in proficiency tests. Each homogenized sample was weighed (20 ± 5 mg) and dissolved in a 10 mL internal standard solution. Then, 1 mL of this solution was transferred to a glass vial, sealed, and subjected to chromatographic analysis. GC–MS analysis (7890A-5975C VL MSD or 7890B-5977B MSD, Agilent Technologies, Santa Clara, CA, USA) was performed for identification based on comparison with in-house libraries (retention time and spectra). An Agilent DB5-MS column (15.0 m × 250 μm × 0.25 μm) was used with helium as the carrier gas at constant pressure with retention time locking. The oven temperature was initially set at 100 °C and then increased to 325 °C. A volume of 1 μL was injected in split mode with a split ratio of 40:1. The run time was 14.25 min. MSD Chemstation E.02.00 SP2 software (Agilent Technologies, Santa Clara, CA, USA) was used for data retrieval.

A miniaturized portable potentiostat (EmStatBlue, PalmSens, Houten, The Netherlands), a handheld Raman spectrometer (Bruker Bravo, Bruker Optik GmbH, Ettlingen, Germany), and a compact ATR-FTIR (Attenuated Total Reflectance Fourier-transform infrared) spectrometer with a diamond crystal (Bruker Alpha II, Bruker Optik GmbH, Ettlingen, Germany) were also used to test the seized samples. For better visualization and comparison, some of the recorded voltammograms were baseline-corrected using the “Moving average” option available in both NOVA1.11 and the PSTrace 5.7 software. In the case of the ATR-FTIR spectrometer, the spectra were recorded from 4000 to 500 cm^−1^ with a resolution of 4 cm^−1^. Each spectrum was an average of 24 scans. Both an in-house spectral MIR library, consisting of reference materials for illicit drugs, and commercial libraries (Bruker; Merck; S.T. Japan, Tokyo, Japan) were used for library matching and identification. The Bravo handheld Raman device has a spectral range of 3200–300 cm^−1^ with a resolution of 10–12 cm^−1^. Two excitation lasers are used with wavelengths of 785 nm and 852 nm. The available libraries are the TICTAC Raman Drug Library and the Forensic Raman Spectra Database. The data interpretation and graph representation were performed using the Microsoft Excel Spreadsheet Software (Microsoft 360) and the OriginPro 8.5 software (OriginLab, Northampton, MA, USA).

(b) Tap water samples were diluted in a 1:1 ratio with a PBS solution containing 20 mM of both K_2_HPO_4_ and KH_2_PO_4_ + 100 mM KCl that was adjusted to pH 12 with NaOH. Then the samples were spiked with MAP to reach a concentration of 0.5 mM. Wastewater samples: were diluted in a 1:1 ratio with PBS of pH 12 (containing 20 mM of both K_2_HPO_4_ and KH_2_PO_4_ + 100 mM KCl) and were then spiked with MAP to reach a concentration of 0.5 mM.

## 3. Results and Discussion

The main purpose of the study was to develop a simple and fast analytical method for the selective and sensitive direct electrochemical detection of the illicit drug MAP from real samples (samples confiscated from the street and water samples). For this, several screen-printed electrode platforms were tested, as well as different experimental conditions. All optimization data are presented and discussed further, with the results also being compared with data from the literature obtained for the detection of MAP. This comparison aims to highlight the advantages and limitations of the analytical method proposed in this study. Moreover, the validation of the method on real samples was done by comparing the results with those obtained with conventional methods used in the current analysis, in this, case GC-MS, GC-FID, FTIR, and Raman (portable devices were used for the FTIR and Raman analyses).

### 3.1. The Electrochemical Characterization of Methamphetamine on Different Platforms by CV

**Figure 1:**
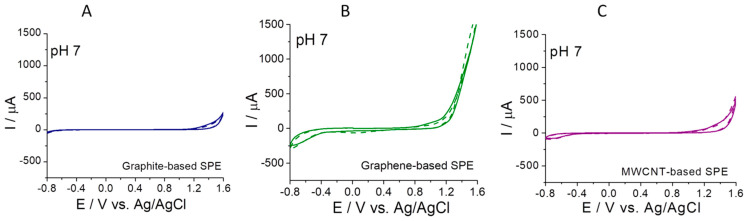

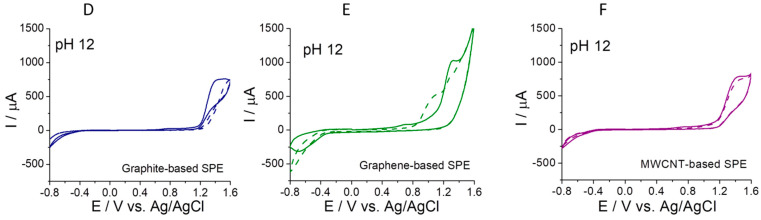
CVs registered for 0.5 mM MAP prepared with: 20 mM PBS of pH 7 (**A**–**C**) and pH 12 (**D**–**F**) on different types of electrodes: graphite-based screen-printed electrodes—(blue); graphite-based screen-printed electrodes modified with—GPH (green); and graphite-based screen-printed electrodes modified with MWCNTs (purple). The dashed lines correspond to the blank tests, recorded on each type of electrode in the absence of MAP. (Experimental parameters for CV tests: potential window from −0.8 V to 1.6 V with a scan rate of 100 mV/s).

CV and MAP solutions prepared in PBS at pH 7 and pH 12 were used to select the most suitable electrode platform and the best pH conditions for the direct detection of this illicit drug. Screen-printed planar electrochemical cells based on graphite screen-printed electrodes, as such or functionalized with graphene (GPH) or multiwalled carbon nanotubes (MWCNTs), were tested. As can be observed from Figure 1, the signal corresponding to the direct electrochemical oxidation of MAP is better highlighted on GPH-functionalized screen-printed electrodes when tested in a pH 12 phosphate buffer medium.

This observation is important given that the same platform and electrolyte have also been selected for other illicit drugs [20], making it an important step in the development of a portable array of sensors that can simultaneously detect several drugs of abuse, each on another electrode in the array but using the same electrolyte. On the graphite electrode functionalized with GPH, the oxidation peak is observed at a potential of about 0.7 V, while on other types of electrodes, the signal position is slightly shifted towards higher values, but the value of the peak current intensity is very small, making it very difficult to compare.

### 3.2. Influence of the Platform Composition and the pH of the Electrolyte

**Figure 2:**
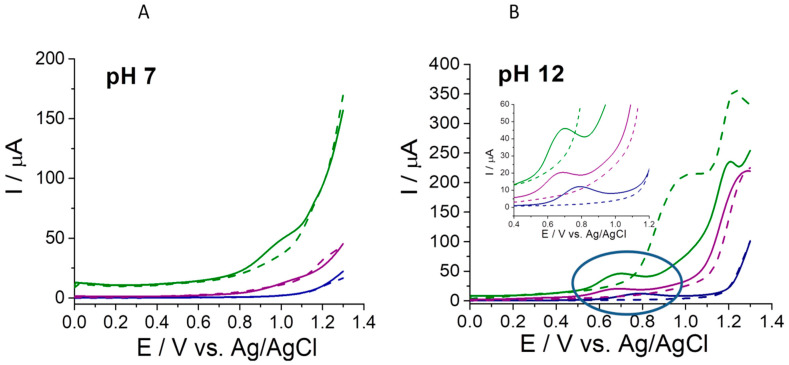
SWVs registered for 0.5 mM methamphetamine prepared with 20 mM PBS of pH 7 (**A**) and 20 mM PBS of pH 12 (**B**) on different types of electrodes: graphite-based screen-printed electrodes (solid blue), graphite-based screen-printed electrodes modified with GPH (solid green), and graphite-based screen-printed electrodes modified with MWCNTs (solid purple). The dashed lines correspond to the blank tests recorded on each type of electrode in the absence of methamphetamine. The graph inserted in Figure (**B**) represents detail for the part of the voltammogram where the electrochemical oxidation signal of methamphetamine is observed. (Experimental parameters for SWV tests: potential window from 0 to 1.3 V, step potential of 5 mV, scan rate of 100 mV/s, amplitude of 25 mV, and a frequency of 10 Hz).

The electrochemical behavior of MAP was evaluated via CV. The influence of pH on the behavior of a 0.5 mM MAP solution was first assessed using CV in the pH range of 7 to 12, but no analytical signal was recorded on any of the tested platforms except at higher pH. Since CV is not a very sensitive method, SWV was used to compare the performance of the three electrode platforms tested. SWV tests were performed, and the results obtained at both pH 7 and pH 12 are presented in Figure 2 and Table 1. It was also observed that the oxidation signal of MAP shows maximum intensity at pH 12 on all tested platforms, but the highest signal was obtained using GPH, so the subsequent optimization tests for the selection of the working pH were carried out on GPH based platforms. The increased sensitivity of the SWV technique compared to CV allowed, in the case of electrodes functionalized with GPH, to observe a small oxidation signal even at pH 7, with the intensity increasing significantly from 1.34 μA at pH 7 to 23.56 μA at pH 12 (Table 1). Simultaneously with the increase in the current intensity, a cathodic shift of the peak potential from 0.985 V to 0.674 V was also observed. This behavior suggests the involvement of an equal number of protons and electrons in the electro-oxidation mechanism of MAP, as previously reported [9,21].

**Figure 3:**
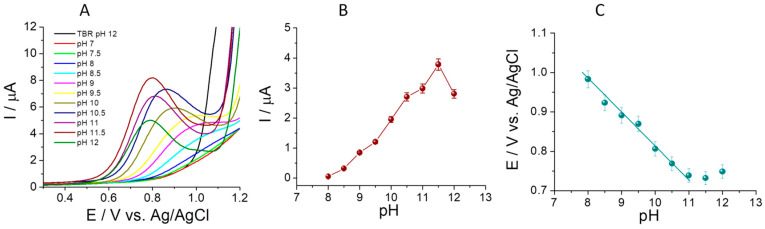
(**A**) SWVs registered on GPH based screen-printed electrodes for 100 μM MAP prepared with BR buffer of different pH. (**B**) Variation of the electrochemical oxidation current of MAP (I_ox_) (wine bullet), and (**C**) of the electrochemical oxidation peak potential of MAP (E_p,ox_) (dark cyan bullet) with the pH value of the electrolyte. (Experimental parameters for SWV tests: potential window from 0 to 1.3 V, step potential of 5 mV, scan rate of 100 mV/s, amplitude of 25 mV, and a frequency of 10 Hz).

The influence of pH on the behavior of a 100 μM MAP solution was tested using SWV in the pH range of 7 to 12 (Figure 3A). The highest signal intensity was observed at a very alkaline pH, as expected, which agrees with the structure of the compound and with other literature data. The effect of pH on the oxidation peak current intensity (I_ox_) of MAP was also studied using 100 μM solutions of the drug in the pH range from 7 to 12, prepared with BR buffer (Figure 3B).

The I_ox_ value (μA) increases spectacularly with the increase in pH from 7 to 11.5, the pH at which the maximum signal is recorded, while at pH 12, the value of the peak current decreases slightly. For further studies, pH 12 was chosen considering that it is desired to integrate the sensor on an array of electrochemical sensors for the simultaneous detection of six illicit drugs, and for the other selected illicit drugs, pH 12 proved to be optimal [20]. This pH value is similar to those reported in other studies for the electrochemical oxidation of MAP on other working electrodes such as graphite [9] or nanodiamond-derived carbon nano-onions decorated with silver nanodendrites [16]. The SWVs in Figure 3A illustrate that the oxidation signal of MAP slightly shifts towards more negative potential values as the pH increases. The oxidation peak potential (E_p,ox_) is linearly dependent on the pH value (Figure 3C) under a regression equation of E_p,ox_ (V) = −0.061 pH + 1.619 (R^2^ = 0.988), with the value of the slope very close to that of the Nernst equation. This suggests that the electrochemical transformation mechanism of MAP on the GPH-based sensor involves an equal number of protons and electrons [1,21].

**Scheme 1:**
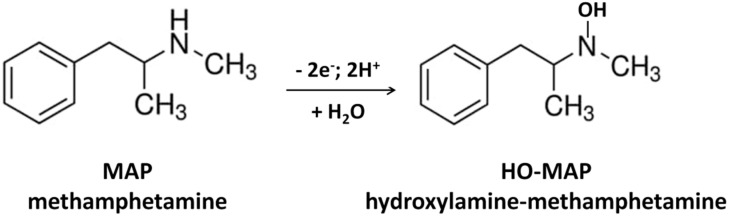
The mechanism proposed for the electrochemical oxidation of MAP in alkaline conditions.

The effect of pH on the peak potential can be explained by considering the theoretical pKa value of MAP, which is 9.5, indicating the predominant presence of its deprotonated form in the analyzed solution. It can be seen in Figure 1 that MAP undergoes irreversible electrochemical transformation [1], a process for which the most predictable mechanism that can be imagined is based on the oxidation of the secondary amine in the MAP structure that occurs more readily in alkaline conditions than in acidic conditions, which aligns with the pKa value of MAP (pKa = 9.5) [21,22]. This phenomenon occurs because the oxidative process is facilitated by the abstraction of an electron from the lone pair of electrons on the amino-nitrogen atom, confirming that the oxidation site of MAP is located on the secondary amino group. The oxidation mechanism of the secondary amino group involves the formation of hydroxylamine through a process that involves a two-step reaction and requires two protons and two electron transfers, as illustrated in Scheme 1. The same mechanism was previously reported by Lee et al. [18]. Based on both the lowest peak potential and the highest I_ox_ values, pH 12 and GPH electrodes were determined to be the optimal conditions for further experiments.

**Figure 4:**
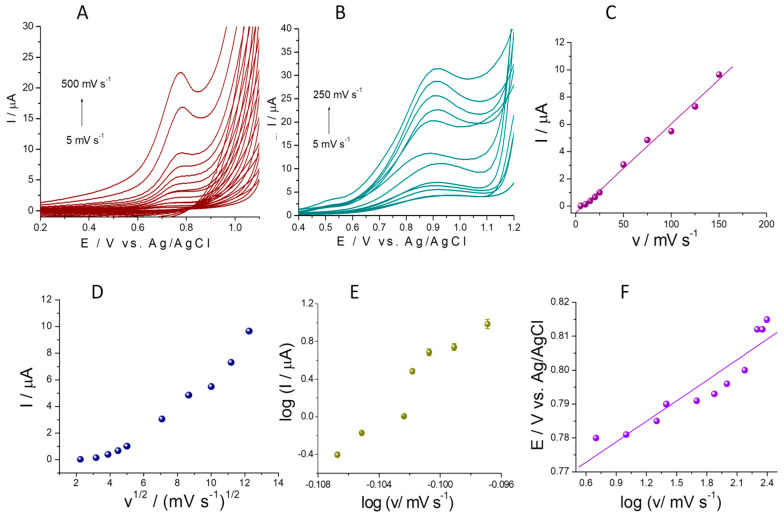
(**A**) CVs registered for 0.5 mM MAP prepared with 20 mM PBS of pH 12 on graphite-based screen-printed electrodes modified with GPH at different scan rates between 5 and 500 mV/s. (**B**) SWVs registered for 100 μM MAP prepared with 20 mM PBS of pH 12 on GPH at different scan rates between 5 and 250 mV/s. (**C**) The current intensity variation with increasing scan rate (from 5 mV s^−1^ to 150 mV/s) (purple bullets). (**D**) with the square root of the scan rate (blue bullets). (**E**) The logarithmic current intensity responds with increasing logarithmic scan rate (dark yellow bullets). (**F**) The variation of the oxidation peak potential with the logarithmic scan rate (violet bullets).

For the elucidation of the kinetics involved in the MAP electrochemical oxidation on the GPH-based sensor, both CV and SWV optimized procedures were applied in the presence of 100 μM MAP solution in 20 mM PBS (pH 12) at the different scan rates (Figure 4A,B). By increasing the scan rate from 5 mV to 500 mV/s for CV or 250 mV/s for SWV, respectively, the intensity of the oxidation peak (I_ox_) increased. A linear variation was obtained for the variation of I_ox_ with the scan rate (Figure 4C) (I_ox_ (μA) = 0.065 v (mV/s) − 0.482), with an excellent correlation coefficient of R^2^ = 0.9915, while for the representation of I_ox_ dependence on the square root of the scan rate, the correlation was modest, at only R^2^ = 0.9606 (Figure 4D). This suggests an electrochemical oxidation process governed by the adsorption of the analyte on the electrode surface, while diffusion and other specific interactions on the GPH platform are negligible. The variation of the logarithm of peak currents with the logarithm of the scan rate was also determined (Figure 4D), and the acceptable correlation (R^2^ = 0.920) confirms that the oxidation of MAP was controlled by adsorption phenomena. This behavior is different from the kinetics reported in other studies [1,9], where on other electrode surfaces it was established that diffusion is the determined step in the mechanism. The change in mechanism in the present study, compared to other studies, may be due to the properties of the GPH nanomaterial on the electrode, which can generate adsorption phenomena for the tested analyte. A linear variation was observed for the oxidation peak potential (E_pox_) with the logarithm of the scan rate using the following equation: E_pox_ (V) = 0.102 log (v (mV/s)) + 0.761; R^2^ = 0.904. The Tafel slope was calculated as 0.187 V based on the slope of the above-mentioned equation, while for 1-α the calculated value was 0.24, which confirms the previously proposed mechanism for the electrochemical oxidation of MAP (Scheme 1) and the literature [21].

### 3.3. Analytical Performance for MAP Detection

**Figure 5:**
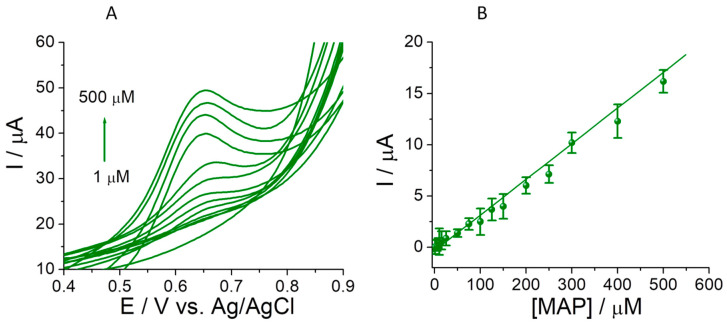
(**A**) SWVs registered for different concentrations of MAP from 1 μM to 500 μM prepared with 20 mM PBS of pH 12 on GPH. (**B**) Calibration curve for MAP electrochemical oxidation on graphite-based screen-printed electrodes with graphene. Error bars correspond to the standard deviation calculated for three different tests on each concentration. (Experimental parameters for SWV tests: potential window from 0 to 1.3 V, step potential of 5 mV, scan rate of 100 mV/s, amplitude of 25 mV, and a frequency of 10 Hz).

In the development of the electroanalytical methodology, the optimization of SWV experimental parameters represents a crucial step. Thus, for the quantification of MAP, the following parameters were optimized: step potential, scan rate, amplitude, and frequency to determine the optimal experimental setup. Experiments were performed on the GPH platform in the presence of 0.5 mM of MAP prepared in PBS pH 12. One parameter was varied while the others were kept constant. The most suitable SWV parameters obtained for MAP detection were a step potential of 5 mV, a scan rate of 100 mV/s, an amplitude of 25 mV, and a frequency of 10 Hz. The optimized procedure was applied to assess the dependence between the analytical signal and its concentration and to draw the calibration curve. Analytical parameters were determined from the obtained SWV curves after spiking the buffer solution (in our case, a 20 mM PBS solution of pH 12) with known volumes of the MAP standard solution. A linear increase in the oxidation current of MAP with the concentration was obtained in the range from 1 to 500 μM. Overlaid voltammograms for all tested concentrations are shown in Figure 5A, while the calibration curve is shown in Figure 5B. The points plotted in the calibration curve represent the mean of at least three tests for each concentration, and the error bars represent the standard deviation calculated for each point. The equation that characterizes the dependence between the recorded current and the MAP concentration is I_ox_ (μA) = 0.031 ± 0.1 × [MAP (μM)] − 0.076 ± 0.22, with a correlation coefficient of R^2^ = 0.9925 and an average relative standard deviation (RSD) of 3.48% over the entire concentration range tested. A LOD of 300 nM was estimated based on the signal-to-noise ratio of 3 (S/*n* = 3), a LOQ of 1 μM was estimated based on S/*n* = 10, and the sensitivity was 30.6 nA nM^−1^.

The window of potential for the detection of MAP was established based on the peak potential values obtained during the calibration. A 5% error was taken into account for the variations generated by changes in temperature. Thus, it was established that the signal of MAP could be detected by using the optimized sensor in the potential window from 0.61 V to 0.73 V.

In Table 2, some examples of MAP detection methods, sensors, and devices are presented for comparative purposes. Analytical parameters are presented (dynamic range, LOD, recoveries, etc.), as well as details regarding the method and type of real samples in which the presence of the illicit drug was tested. It can be seen that most of the detection methods present a better detection limit than the one obtained in the present study, but this aspect is not relevant because our sensor is designed to be useful for simple and fast testing of real samples captured from the street, where the drug content is high and the test solutions obtained have mM concentrations of MAP. The levels of MAP in human blood were found to be between 0.03 mg/L (160 nM) and 0.41 mg/L (2.19 μM) [23]. However, the analytical parameters are better than those obtained on a similar platform but based on unmodified graphite [9], thus justifying the use of graphene for the functionalization of the working electrodes. The positive outcomes of our optimized detection strategy can be attributed to the utilization of GPH as a distinctive nanomodifier. This modifier serves to enhance the surface area of the graphene screen-printed electrodes, thereby amplifying the electrocatalytic reaction. As a result, the overall efficiency of the electrochemical process has significantly improved.

The inter-assay reproducibility of the nanostructured platform (inter-assay stability) was investigated with six sensors, recording and comparing the voltammograms in the presence of 0.5 mM MAP in PBS of pH 12 under the same experimental conditions. An RSD of 3.61% was obtained, proving the reproducibility of the sensor platform and the good repeatability (precision) of the method. Next, three successive tests were performed on a single sensor, with a new analyte solution for each test, to test the stability of the analytical signal upon retesting and the possibility of reusing the sensors. It was observed that the signal of the illicit drug decreased by approximately 16% after the three tests, so it is recommended that the sensor be reused only after an electrochemical regeneration of the electrode surface by cycling in 0.1 M H_2_SO_4_. This aspect is also important from the perspective of avoiding the risk of contamination of the real samples analyzed by retesting on the same sensor.

### 3.4. Selectivity Tests for MAP Detection

**Figure 6:**
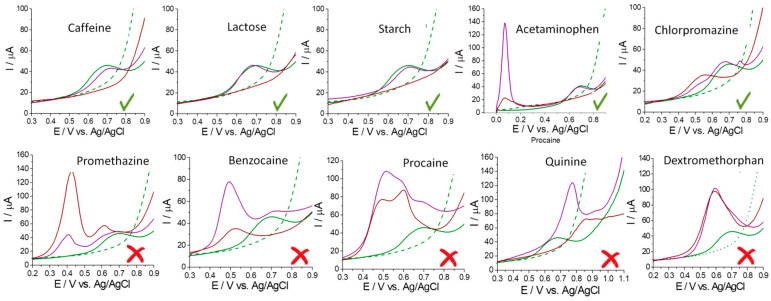
Selectivity study for the detection of MAP in the presence of different adulterants or cutting agents. The overlapped voltammograms represent the blank test in buffer solution (dash green), the test obtained in the presence of 0.5 mM MAP (solid green), the test obtained for 0.5 mM of adulterant/ cutting agent (dark red), and the 1:1 binary mixtures of MAP with these compounds (purple). (Experimental parameters for SWV tests: potential window from 0 to 1.3 V, step potential of 5 mV, scan rate of 100 mV/s, amplitude of 25 mV, and a frequency of 10 Hz). 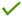 = no interference; 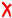 = interference.

The evaluation of the electrochemical behavior of some adulterants was performed on the optimized sensor platform, found alone in solution or in the presence of MAP. Several 1:1 mixtures of MAP with some of the most commonly reported adulterants or cutting agents for this illicit drug were tested. The obtained results are presented in Figure 6.

Ten possible interferents were tested, namely: caffeine, lactose, starch, acetaminophen, chlorpronazine, promethazine, benzocaine, procaine, quinine, and dextromethorphan. It was found that MAP can be tested with good selectivity in the presence of caffeine, lactose, starch, acetaminophen, and chlorpronazine, while the presence of promethazine, benzocaine, procaine, quinine, and dextromethorphan partially hindered the access of the target analyte to the electrode surface for electronic transfer. This can be due to the oxidation peak of the aforesaid adulterants being near or in the same window of potential as the oxidation peak of MAP, generating its shift or suppression.

From the SWV data, the recoveries obtained for MAP in the presence of the ten compounds were calculated by comparing them with the signal obtained at the same concentration for standard solutions of MAP, data included in the calibration curve. The data are presented in Table 3, where recovery values between 10.45% and 102.2% can be observed.

Additionally, binary mixtures of MAP were tested with other amphetamine-type stimulants (i.e., MDMA and AMF) and ephedrine, a precursor of MAP, in a combined ratio of 1:1. Importantly, in the tested potential range, no electrochemical signal was recorded for AMF or ephedrine, this behavior is an agreement with the previously reported literature [31,32]. Excellent recoveries were obtained for MAP in the tested binary mixtures, namely 87.95%, 94.40%, and 99.15% in the presence of AMF, ephedrine, and MDMA, respectively.

### 3.5. Assessment of Real Samples

The practical applicability of the sensor and the optimized detection method was tested by analyzing water samples spiked with MAP and real seized samples provided by the National Institute of Criminalistics and Criminology (NICC) in Belgium (see all the details related to the preparation of real samples for testing and the control methods applied in Section 2.5).

Testing of the electrochemical sensor for MAP detection was done on water samples to which known volumes of MAP stock solution were added, as described in the experimental part. Tap water and wastewater were tested in both situations without any pretreatment applied to the sample, except for the analyte spike with a known amount of MAP. The obtained samples were electrochemically tested with the optimized SWV method, and recoveries were calculated using the equation of the calibration line. The results obtained for spiked water samples are presented in Table 4. Good recovery rates were obtained, and it can be concluded that MAP can be identified from water samples, illustrating the potential of the sensor as a useful tool for environmental analysis.

**Figure 7:**
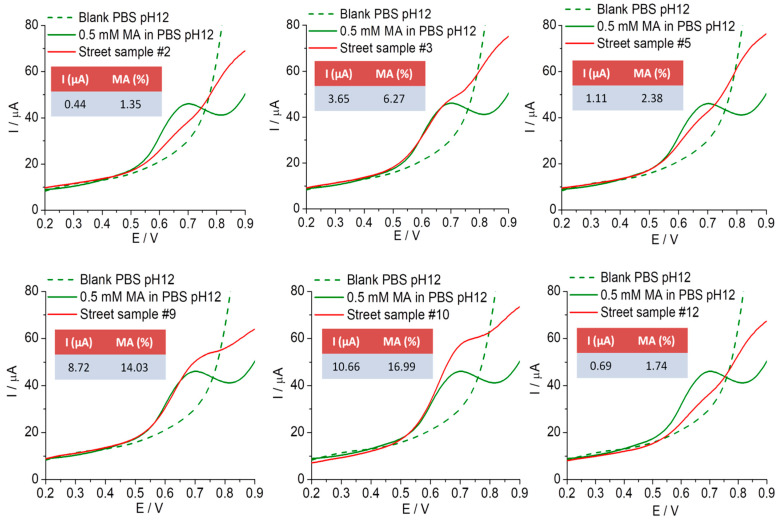
Detection of MAP in powdered street samples. The overlapped voltammograms represent the blank test in buffer solution (dash green), the test obtained in the presence of 0.5 mM methamphetamine (solid green), and the test obtained for the solution containing the confiscated powder (red). (Experimental parameters for SWV tests: potential window from 0 to 1.3 V, step potential of 5 mV, scan rate of 100 mV/s, amplitude of 25 mV, and a frequency of 10 Hz).

In the case of real samples captured from the street, nine samples with different compositions and contents of MAP and three samples containing cocaine were tested. The composition of the drug samples was determined by GC-MS and GC-FID, then the samples were analyzed with the optimized SWV method with a portable Raman and FTIR. For six samples, the voltammograms were plotted and compared to the blank signal recorded in the absence of the drug and the signal obtained in the presence of a MAP standard of known concentration (Figure 7). Each sample was retested at least three times on different GPH-based sensors, and the mean of the oxidation peak current values was used to calculate the illicit drug content in the sample. The electrochemical profile of the seized samples was compared to the electrochemical profile of a 0.5 mM MAP standard solution. This step is important because the presence or absence of MAP can be detected through the presence or absence of the characteristic oxidation peak in the same potential range, and implicitly, the presence of the drug in the analyzed sample can be observed.

The results obtained when testing the twelve samples with the optimized SWV method but also with the previously mentioned control methods are presented in Table 5 and Table 6. It can thus be concluded that the use of the electrochemical method and the GPH-based sensor allowed the detection of eight seized samples out of nine (89%), these being true-positive results. Moreover, true-negative results (100%) were obtained when the optimized sensor based on GPH was tested in the presence of three real samples containing cocaine. The optimized SWV method proved to be more sensitive than the portable Raman device, which allowed the detection of only three samples containing MAP out of nine (33%).

The FTIR device identified nine out of nine (100% true positive rate) real samples containing MAP, and it identified DMSO as an adulterant in five samples, this being in concordance with the GC-MS results. For the validation parameters, the electrochemical detection of MAP on the disposable graphene screen-printed electrodes exhibited a similar performance to the FTIR device (92% vs. 100% accuracy) and a net superior performance to the Raman device (Table 6). Furthermore, these values are in accordance with previous evaluations of electrochemical screening of MAP in seized samples [9].

## 4. Conclusions

In this study, a graphite-based screen-printed platform with a working electrode functionalized with graphene was used for the first time for the elaboration of an electrochemical sensor for methamphetamine. The use of graphene for functionalization proved to be a good strategy for improving the analytical performance of the sensor for the simple and cost-effective direct electrochemical detection of the targeted drug of abuse. Cyclic voltammetry and square wave voltammetry were the electrochemical methods applied for the characterization of the nanostructured platform and for the quantification of MAP. The optimized method is less time-consuming and less expensive than other analytical methods currently applied for the determination of MAP, especially chromatography and hyphenated techniques. In addition, the optimized platform presents the advantages of portability and a detection limit of 5 μM, lower than the previously reported sensor based on graphite screen-printed electrodes, with the potential to be employed by law enforcement agencies for the fast and accurate screening of suspected cargo.

Finally, the optimized method was successfully applied to analyze real samples containing MAP and validate the applicability of the sensor in practical scenarios. The method proved sufficiently selective for the detection of MAP not only from samples mixed with other drugs or adulterants but also from real samples with complex matrices such as tap water, wastewater, and seized samples captured from the street.

By evaluating the performance of our nanostructured platform using real samples, we obtained valuable insights into its real-world viability. It can be assumed that it can be easily adapted for the detection of other illicit drugs and has the potential for deployment in drug detection applications.

## Figures and Tables

**Table 1:** The influence of the working electrode configuration and pH of the electrolyte solution on the electrochemical oxidation of MAP.

WE Material	pH	I_ox_ (μM)	E_ox_ (V)
graphite	7	-	-
GPH		1.34	0.985
MWCNT		-	-
graphite	12	4.14	0.787
**GPH**		**25.76**	**0.674**
MWCNT		8.66	0.665

With bold: the experimental parameters selected for the study; WE: working electrode.

**Table 2:** Electrochemical detection methods of MAP.

No.	Method	Working Electrode	LOD (μM)	Linear Range (μM)	Real Samples/Recovery (%)	Time (s)	Ref.
1.	SWV	MWCNTs/Au-NPs/SH (CH2)_3_-Si-SiO^2^@Fe_3_O_4_ GCE	0.016	0.05–50	urine/101–111	-	[24]
2.	FFT-SWV	MIPs-MWCNTs CPE	0.83 × 10^−3^	0.01–1 3–100	urine/94.2–96 serum/92.8–104.6	>20	[2]
3.	spilt SWV	C-screen-printed electrodeswith a mediator	2.68	0–33.5	-	55	[25]
4.	DPV	Polypeptide GCE	87.58	67–670	urine/98.54–105.99 serum/94.43–103.58 saliva/90.03–93.06	-	[26]
5.	ECL	Ru(bpy)_3_]^2+^–Nafion composite GCE	0.05 × 10^−3^	0.005–1000	-	15	[6]
6.	SWSV	AuNPs/MWCNTs screen-printed electrodes	0.006	0.2–0.1 3.0–50	-	>200	[7]
	EIS		0.3 × 10^−3^	(1.15–2.69) × 10^−3^	-		
7.	DPV	PPGE	0.05	0.075–54	street sample/99.71–102.57 serum/96.91–100.5 urine/102.40–95.64	>600	[27]
8.	ECL	Imunosensor	0.002	0.013–3.35	serum/90–106	-	[28]
9.	DPV	BDDE	0.05	0.07–80	human urine/93.4–97.6	-	[1]
10.	Amp	anti-MA/nanoAu/MPS/PB/LC Au electrode	7.5 × 10^−3^	0.01–5	human blood/96.9–104.2	-	[29]
11.	EIS	Immunosensor	0.677 × 10^−7^	(1.34–13.4) × 10^−7^	serum/80.8–104	-	[30]
12.	SWV	C-screen-printed electrodes	16.7	50–2500	street samples/-		[9]
13.	SWV	GPH-screen-printed electrodes	0.3	1–500	wastewater/82.01 tap water/89.72 powder/-	5	This work

Amp = amperometry; anti-MA/nano-Au/MPS/PB/LC Au electrode = -methamphentamine antibody-gold nanoparticles-(3-mercaptopropyl trimethoxysilane)-persian blue-L-cysteine modified gold electrode; AuNPs/MWCNTs = screen printed electrode modified with multi-walled carbon nanotubes and gold nanoparticles; BDDE = boron doped diamond electrode; DPV = differential pulse voltammetry; ECL = electrochemiluminescence; EIS = electrochemical impedance spectroscopy; FFT-SWV = fast Fourier transform square wave voltammetry; GPH = graphene; MIPs-MWCNTs-CPE = carbon paste electrode modified with MIPs and multi-walled carbon nanotubes; MWCNTs/Au-NPs-SH (CH2)_3_-Si-SiO_2_@Fe_3_O_4_ GCE = multi-walled carbon nanotubes-gold nanoparticles linked to nanomagnetic core shells modified glassy carbon electrode; PPGE= pretreated pencil graphite electrode; C = carbon electrode; SWV = square wave voltammetry; SWSV = square wave stripping voltammetry.

**Table 3:** Variation of the analytical performances of MAP depending on the adulterant molecule.

Pure Drug		Pure Adulterant			Mixture		MAP Recovery (%)
E (V)	I (µA)	Name	E (V)	I (µA)	E (V)	I (µA)	
0.650	15.91	Caffeine	-	-	0.694	14.84	93.27
		Lactose	-	-	0.669	16.26	102.20
		Starch	-	-	0.694	14.85	93.33
		Acetaminophen	0.07	14.95	0.075	150.00	92.45
					0.699	14.71	
		Chlorpromazine	0.538	11.10	0.503	0.33	87.87
					0.664	13.98	
		Promethazine	0.422	104.00	0.407	23.72	68.07
			0.614	13.62	0.664	10.83	
		Benzocaine	0.513	13.36	0.488	48.60	22.63
					0.709	3.60	
		Procaine	0.529	28.00	0.503	58.37	10.45
					0.704	1.66	
		Quinine	0.871	18.15	0.790	55.83	UD *
					0.936	0.55	
		Dextromethorphan	0.584	62.87	0.589	72.09	UD

* UD = undetectable.

**Table 4:** Assessment of methamphetamine in water samples using the developed sensor.

I (µA) of 0.5 mM MA in Standard Solution	I (µA) of 0.5 mM MA in Tap Water Solution	Recovery/RSD (%)	I (µA) of 0.5 mM in Wastewater Solution of MA	Recovery/RSD (%)
16.18	14.52	89.72/2.76	13.27	82.01/14.54

**Table 5:** Testing the electrochemical sensor for MAP detection from real samples captured from the street. Comparison with other methods and devices currently used in the field and in the laboratory.

Sample No.	GC-MS Analysis (Identified Compound)	GC-FID (%)	Electrochemical Sensor E (V)	Portable Raman (Identified Compound)	FTIR (Identified Compound)
#1	MAP + DMSO	81	0.67	dichloromethane	MAP + DMSO
#2	MAP + DMSO	64	0.67	Dichloromethane	MAP + DMSO
#3	MAP	97	0.65	ephedrine HCl	MAP
#4	MAP + DMSO	85	0.73	ephedrine HCl	MAP + DMSO
#5	MAP + DMSO	46	0.7	ephedrine HCl	MAP + DMSO
#6	MAP + DMSO	8.5	0.69	Dichloromethane	MAP + DMSO
#7	MAP	99	0.73	MAP	MAP
#8	MAP	97	0.69	MAP	MAP
#9	MAP	95	0.8/ND	MAP	MAP
#10	Cocaine	87	ND	Cocaine	Cocaine
#11	Cocaine + Caffeine	92	ND	Cocaine	Cocaine
#12	Cocaine + Levamisol	75	ND	Cocaine	Cocaine

**Table 6:** The balance of true positive (TP) and false negative (FN) results obtained for the twelve real samples with all the methods considered in the study.

Sample No.	GC-MS Analysis	Electrochemical Sensor	Portable Raman	Portable FTIR
#1	MAP + DMSO	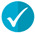	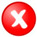	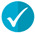
#2	MAP + DMSO	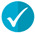	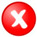	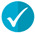
#3	MAP	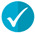	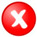	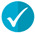
#4	MAP + DMSO	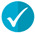	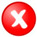	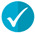
#5	MAP + DMSO	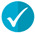	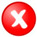	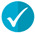
#6	MAP + DMSO	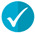	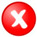	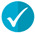
#7	MAP	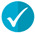	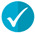	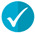
#8	MAP	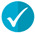	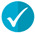	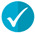
#9	MAP	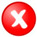	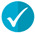	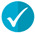
#10	Cocaine	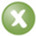	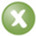	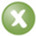
#11	Cocaine + Caffeine	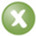	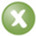	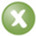
#12	Cocaine + Levamisol	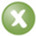	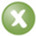	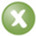
TP (sensitivity)	100% (12)	89% (8)	33% (3)	100% (9)
TN	0% (0)	100% (3)	100% (3)	100% (3)
FN	0% (0)	11% (1)	67% (6)	0% (0)
Accuracy	100%	92%	50%	100%

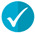
 true positive test for MAP; 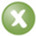 true negative tests for MAP; 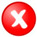 false negative test for MAP; TP—true positive; FN—false negative; TN—true negative.

## Data Availability

Data is contained within the article.
